# Roles of miR-592-3p and Its Target Gene, *TMEFF1,* in the Nucleus Accumbens During Incubation of Morphine Craving

**DOI:** 10.1093/ijnp/pyac004

**Published:** 2022-01-10

**Authors:** Bing Xie, Jingjing Zhang, Chunling Ma, Hailei Yu, Zhiyu Ni, Bin Cong, Di Wen

**Affiliations:** College of Forensic Medicine, Hebei Medical University, Hebei Key Laboratory of Forensic Medicine, Collaborative Innovation Center of Forensic Medical Molecular Identification, Research Unit of Digestive Tract Microecosystem Pharmacology and Toxicology, Chinese Academy of Medical Sciences, Hebei Province, Shijiazhuang, PR China; College of Forensic Medicine, Hebei Medical University, Hebei Key Laboratory of Forensic Medicine, Collaborative Innovation Center of Forensic Medical Molecular Identification, Research Unit of Digestive Tract Microecosystem Pharmacology and Toxicology, Chinese Academy of Medical Sciences, Hebei Province, Shijiazhuang, PR China; College of Forensic Medicine, Hebei Medical University, Hebei Key Laboratory of Forensic Medicine, Collaborative Innovation Center of Forensic Medical Molecular Identification, Research Unit of Digestive Tract Microecosystem Pharmacology and Toxicology, Chinese Academy of Medical Sciences, Hebei Province, Shijiazhuang, PR China; College of Forensic Medicine, Hebei Medical University, Hebei Key Laboratory of Forensic Medicine, Collaborative Innovation Center of Forensic Medical Molecular Identification, Research Unit of Digestive Tract Microecosystem Pharmacology and Toxicology, Chinese Academy of Medical Sciences, Hebei Province, Shijiazhuang, PR China; School of Basic Medical Science, Hebei University, Hebei Province, Baoding, PR China; College of Forensic Medicine, Hebei Medical University, Hebei Key Laboratory of Forensic Medicine, Collaborative Innovation Center of Forensic Medical Molecular Identification, Research Unit of Digestive Tract Microecosystem Pharmacology and Toxicology, Chinese Academy of Medical Sciences, Hebei Province, Shijiazhuang, PR China; College of Forensic Medicine, Hebei Medical University, Hebei Key Laboratory of Forensic Medicine, Collaborative Innovation Center of Forensic Medical Molecular Identification, Research Unit of Digestive Tract Microecosystem Pharmacology and Toxicology, Chinese Academy of Medical Sciences, Hebei Province, Shijiazhuang, PR China

**Keywords:** Conditioned place preference, incubation, miR-592-3p, morphine craving, nucleus accumbens, *TMEFF1*

## Abstract

**Background:**

Prolonged forced abstinence from morphine can increase cue-induced cravings for the drug, contributing to a persistent vulnerability to relapse. Previous studies have identified the implications of aberrant microRNA (miRNA) regulation in the pathogenesis of morphine addiction, but the changes in miRNA expression during the incubation of morphine craving are still unknown.

**Methods:**

Nucleus accumbens (NAc)-specific altered miRNA transcriptomics was determined in a mouse model of cue-induced incubation of morphine craving following a next-generation sequencing method and verified by RT-qPCR. Bioinformatics analysis was performed to predict the target gene of selected miRNA, and the protein expression of the target gene was detected by western blot. A dual-luciferase assay was performed to confirm the binding sites, and gain- and loss-of-function strategy was applied to understand the mechanism of miRNA and its target gene.

**Results:**

The miR-592-3p observed to be downregulated in the NAc core was linked to the incubation of morphine craving, and a dual-luciferase assay was performed to confirm the binding sites of miR-592-3p in its target gene, tomoregulin-1 (*TMEFF1*). Also, gain- and loss-of-function analyses revealed that the inhibition of miR-592-3p expression in the NAc core negatively regulated *TMEFF1* expression, thereby enhancing the incubation of morphine craving; however, the overexpression of miR-592-3p in the NAc core resulted in a decreased expression of *TMEFF1*, thereby reducing the incubation of morphine craving.

**Conclusion:**

Our findings demonstrated that miR-592-3p can improve the incubation of morphine craving by targeting *TMEFF1*, and thus, it holds a therapeutic potential to inhibit opioid craving.

**Significance Statement**

The current study, focused on the epigenetic mechanism of the incubation of morphine craving, address 2 questions: (1) How is the expression of key microRNAs (miRNAs), as epigenetic modulators, altered during the cue-induced incubation of morphine craving? (2) Which is/are the target gene(s) and what is/are the regulatory mechanism(s) of gene(s) in response to the candidate miRNAs? Gain- and loss-of-function strategy analyses revealed that miR-592-3p can improve the incubation of morphine craving by targeting TMEFF1. This finding addresses a gap in the current literature regarding the molecular mechanisms engaged in the incubation of morphine craving. Future research into the upstream mechanism of miR-592-3p is needed to shed light on the molecular regulatory network on the incubation of morphine craving. Ultimately, this line of research advances knowledge that may hold a therapeutic potential to inhibit opioid craving.

## Introduction

In the ongoing fight against drug addiction, one major challenge that must be overcome is the high rate of relapse or returning to drug use after periods of withdrawal (Hunt et al., 1971). One frequent trigger for relapse is exposure to cues and contexts associated with drugs, even after prolonged periods of abstinence (Gawin and Kleber, 1986; Ehrman et al., 1992). Researchers have previously demonstrated that cue-induced drug craving and seeking increases progressively during abstinence, termed “incubation of drug craving” (Grimm et al., 2001; Lu et al., 2004). In the past decade, several studies have established some neural mechanisms underlying the incubation of drug cravings after forced abstinence, having focused primarily on understanding cravings for cocaine, heroin, and methamphetamines (Wolf, 2010; Pickens et al., 2011; Loweth et al., 2014). Emerging evidence supports the idea that epigenetic changes are important mechanisms underlying the concept of addiction and the neurobiological response to addictive substances (Nielsen et al., 2012; Browne et al., 2020). Massart et al. demonstrated a role for nucleus accumbens (NAc) DNA methylation, and downstream targets of DNA demethylation, in incubation of cocaine craving (Massart et al., 2015). Another study revealed that NAc miR-181a and methyl CpG binding protein 2 (MECP2) contribute to incubation of heroin craving (Xu et al., 2021). However, although several brain regions and molecular mechanisms are engaged in the incubation of morphine craving, the underlying epigenetic mechanisms remain unknown at this time. Understanding the epigenetic mechanism of the incubation of morphine craving is likely to have significant implications for preventing drug relapse.

MicroRNAs (miRNAs), as epigenetic modulators, are small endogenous noncoding RNAs that negatively regulate gene expression at the posttranscriptional level by binding to the “seed regions” found at the 3′-untranslated regions (3′-UTRs) of their target messenger RNA (mRNA) (Mahmoudi and Cairns, 2017). To date, they have been suggested to be a group of regulators that play roles in a broad range of biological processes, such as cell proliferation, differentiation, apoptosis, and cycle regulation (Mansini et al., 2018; Nowakowski et al., 2018; Bhaskaran et al., 2019); furthermore, miRNAs appear to be abundant in the nervous system, acting as regulatory molecules in processes such as neurogenesis, synapse formation, and neuroplasticity development (Ziats and Rennert, 2014; Song, 2020; Yoshino et al., 2021). Recently, changes in several specific miRNA expressions that may influence the interactions between miRNAs and their targets during addiction were identified by several studies (Dreyer, 2010; Pietrzykowski, 2010; Bali and Kenny, 2013; Smith and Kenny, 2018). Notably, miR-133b is expressed in midbrain dopaminergic neurons and regulates the dopamine transporter and the production of tyrosine hydroxylase. In contrast, miR-212 influences the production of the striatal brain–derived neurotrophic factor and synaptic plasticity following cocaine intake (Bali and Kenny, 2013). Some investigations have suggested specific miRNAs to be key regulators in drug addiction that might also act as valuable targets for the development of more efficient therapies and the prevention of relapse (Kenny, 2014; Smith and Kenny, 2018).

The incubation of drug craving is regulated by maladaptive cellular plasticity in several mesolimbic brain regions, including NAc, a central reward–related region in which several pathways involved in reward and motivation converge (Sesack and Grace, 2010). Previous studies have reported that altering the miRNA expression in the NAc appears sufficient to attenuate or enhance drug-seeking behaviors (Chandrasekar and Dreyer, 2011). Other research has revealed that miRNAs induced by drug abuse may modify the expression levels of many downstream target genes that control addiction-linked neural mechanisms (Saba et al., 2012). Nevertheless, little is known about miRNAs involved in the incubation of morphine craving and their expression levels.

The current study aimed at addressing 2 questions: (1) How is the expression of key miRNAs altered in the NAc during the cue-induced incubation of morphine craving? and (2) Which is/are the target gene(s) and what is/are the regulatory mechanism(s) of gene(s) in response to the candidate miRNAs? To answer these 2 questions, the cue-induced incubation of the morphine craving model was first established by using the conditioned place preference (CPP) paradigm. Then, the aberrant expression of miRNAs was identified in the NAc tissue by RNA sequencing. The molecular mechanisms of downstream target genes involved in the incubation of morphine craving were investigated by the gain- and loss-of-function strategy. Our study offers evidence concerning the underlying mechanisms of the cue-induced incubation of morphine craving and suggests novel potential targets for preventing relapse.

## MATERIALS AND METHODS

### Animals

Male C57BL/6N mice (body weight: 18–25 g) were acquired from Liaoning Changsheng Co., Ltd. (Liaoning, China) and maintained on a 12-hour-light/-dark reversed cycle with freely available food and water. Each of the experimental procedures was endorsed by the Animal Care and Use Committee of Hebei Medical University and was performed in accordance with the National Research Council Guide for the Care and Use of Laboratory Animals. Morphine sulfate was obtained from Qinghai Pharmaceutical Factory Co., Ltd. (Qinghai, China). Drug concentrations in this study were modified to an appropriate injection volume of 5 mL/kg body weight with saline, and all drug solutions were prepared fresh on the day of their administration.

### Morphine CPP Training

An unbiased, morphine-induced CPP was performed as previously detailed, with slight modifications (Sun et al., 2018). The CPP apparatus was divided into 3 visually and tactually distinct compartments isolated by guillotine doors (JLBeh Soft-tech Co. Ltd., China). In short, the CPP schedule consisted of preconditioning, conditioning, and testing phases. Among these, first, the preconditioning phase (Day 1) established the baseline preference. Mice were first set in the middle chamber without the doors installed for 15 minutes, and their time spent in the 2 side chambers was calculated as a baseline value, referred to henceforth as T0. The mice with a CPP score >150 seconds were excluded. Conditioning phase (Day 2–7) included CPP training. Each mouse was treated for 6 days with alternate injections of morphine (1 mg/kg or 5 mg/kg, i.p.) or saline (1 mg/kg or 5mg/kg, i.p.) at an interval of 6 hours; mice were immediately confined to its drug-paired or saline-paired chamber for 45 minutes. Testing phase involved morphine withdrawal and was labeled as withdrawal day 1 (WD1/Day 8). During the testing phase, the guillotine doors were removed to give the mice free access to the entire apparatus, and the mice were placed in the middle chamber to give them free movement throughout the entire apparatus for 15 minutes; morphine withdrawal occurred on day 14 (WD14/Day 21). Thereafter, 2 post-conditioning tests were performed: test 1 (T1) and test 2 (T2) on day 1 and day 14, respectively, after the last conditioning sessions in a morphine-free state.

### RNA Isolation, RNA Library Preparation, and Sequencing

The NAc tissues (core and shell) from C57BL/6N mice from the 2 groups subjected to the morphine-induced CPP model test 1 (T1) and test 2 (T2) were harvested; 3 samples (6 mice) from each group were used for total RNA isolation. RNA-seq was performed by Novogene Co., LTD (Beijing, China).

#### Library Preparation for Small RNA Sequencing

A total amount of 3 μg total RNA per sample was used as input material for the small RNA library. Sequencing libraries were generated using NEBNext Multiplex Small RNA Library Prep Set for Illumina (NEB, Ipswich, MA, USA) following the manufacturer’s recommendations, and index codes were added to attribute sequences to each sample. PCR amplification was performed using LongAmp Taq 2X Master Mix, SR Primer for Illumina and index (X) primer. DNA fragments corresponding to 140 to approximately 160 bp (the length of small noncoding RNA plus the 3’ and 5’ adaptors) were recovered and dissolved in 8 μL elution buffer. Lastly, library quality was assessed on the Agilent Bioanalyzer 2100 system using DNA High Sensitivity Chips.

#### Clustering and Sequencing

The clustering of the index-coded samples was performed on a cBot Cluster Generation System using TruSeq SR Cluster Kit v3-cBot-HS (Illumia) according to the manufacturer’s instructions. After cluster generation, the library preparations were sequenced on an Illumina Hiseq 2500/2000 platform, and 50-bp single-end reads were generated.

#### Quality Control

Raw data of fastq format were firstly processed through custom perl and python scripts. Then, a certain range of length from clean reads was chosen to conduct all the downstream analyses.

#### Reads Mapping to the Reference Sequence

The small RNA tags were mapped to reference sequence by Bowtie (Langmead et al., 2009) without mismatch to analyze their expression and distribution on the reference.

#### miRNA Editing Analysis

Position 2 to approximately 8 of a mature miRNA was considered the seed region, which was highly conserved. The target of a miRNA might differ with changing nucleotides in this region. In our analysis pipeline, miRNA that might have a base edit could be detected by aligning all the sRNA tags to mature miRNA, allowing 1 mismatch.

#### miRNA Family Analysis

We explored the occurrence of miRNA families identified from the samples in other species. In our analysis pipeline, known miRNA used miFam.dat (http://www.mirbase.org/ftp.shtml) to look for families; novel miRNA precursor was submitted to Rfam (http://xfam.org/) to look for Rfam families.

#### Differential Expression of miRNA

Differential expression analysis of 2 conditions/groups was performed using the DESeq R package (1.8.3). *P* values were adjusted using the Benjamini and Hochberg method. The average reads of the samples were 17 546 185 (supplementary Table 1). The corrected *P* value of .05 was set as the threshold for significantly differential expression by default. Data information is shown in supplementary Table 1 (RNA-seq data accessible at NCBI GEO database, accession GSE192888).

### Quantitative Reverse Transcription-Polymerase Chain Reaction (qRT-PCR) Analysis

**Figure 1.**
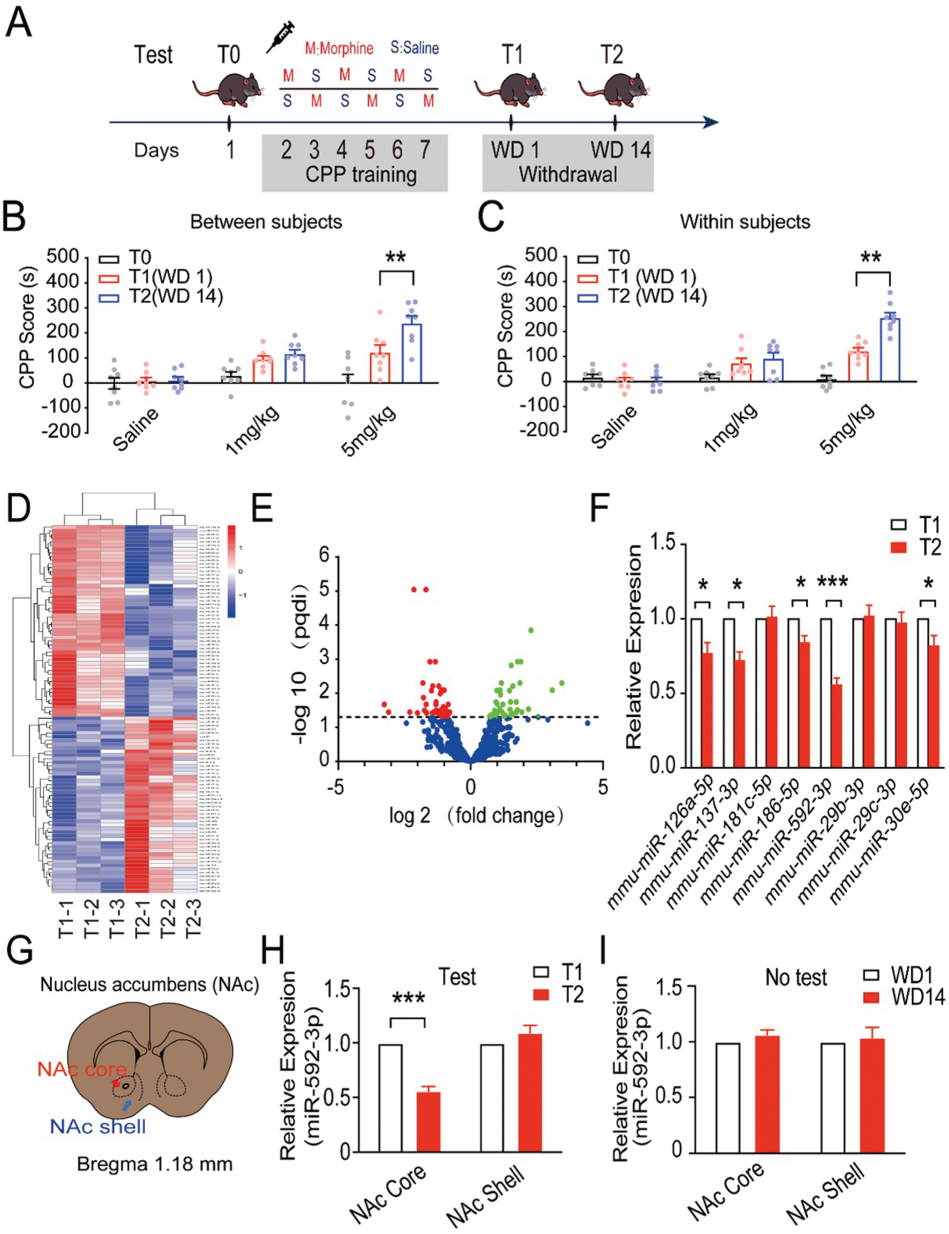
Expression of miRNAs involved in the cue-induced incubation of morphine craving. (A) An illustrated representation of the morphine conditioned place preference (CPP) training timeline. (B) The influence of morphine dose and test day on morphine CPP score. The between-subjects assessment was performed on 9 groups of mice (n = 8 per group), where each group was tested on a single withdrawal day. **P < .01 vs T1 group. (C) Within-subjects assessments were performed on 3 groups of mice (n = 8 per group), and each group was separately tested at withdrawal 1 (WD1) and WD14. **P < .01 vs T1 group. (D) The heatmap shows clusters of miRNAs differentially expressed between WD1 and WD14 samples. (E) A volcano plot to show the significantly differentially expressed miRNAs in WD1 as compared with WD14. Green dots show overexpressed miRNAs and red dots show underexpressed miRNAs. (F) The expression of 8 downregulated miRNAs in the nucleus accumbens (NAc) on WD1 and WD14 (n = 3). *P < .05; ***P < .001. (G) Two subdivisions of the NAc: core (NAcC) (red arrows) and shell (NAcSh) (blue arrows). (H) The expression of miR-592-3p in NAcC or NAcSh with test, ***P < .001 vs T1 group. (I) The expression of miR-592-3p in NAcC or NAcSh without test.

The NAc core was perforated with an approximately 600-μm micro-punch to obtain brain sections (BT1.0; The Harris Products Group, Mason, OH, USA), and the NAc shell was dissected using a needle based on the mouse brain atlas (Fig. 1G). The total RNA from the brain tissue was extracted by using the miRNAeasy Mini Kit (QIAGEN, USA). Total RNA (2.5 μg) was incubated with 10 U/μg of RNase R (Geneseed Biotech, China) at 37°C for 10 minutes. A total of 1.0 µg of RNA was used for RT using the PrimeScript RT Reagent Kit (TaKaRa, China) and miScript II RT Kit (Qiagen). qRT-PCR analysis was performed with TB Green Premix Ex Taq II (TaKaRa) and the miScript SYBR Green PCR Kit (Qiagen) based on the company’s directions. All reactions were set up in 20-μL volumes on a QuantStudio Flex (Applied Biosystems). The *GAPDH* mRNA and *U6 snRNA* were utilized as reference genes to analyze mRNA and miRNA, respectively. The 2^−ΔΔCt^ method was utilized to calculate the relative expression levels of mRNAs and miRNAs.

### Bioinformatics Analysis

The putative miR-592-3p target genes were predicted by TargetScan (http://www.targetscan.org/vert_72/) and miRanda (https://bioweb.pasteur.fr/packages/pack@miRanda@3.3a). Gene-ontology (GO) enrichment analysis was performed to identify the target gene candidates of miR-592-3p; meanwhile, KEGG analysis was conducted to predict the pathways of the target gene candidates. GO enrichment analysis was used on the target gene candidates of differentially expressed miRNAs (“target gene candidates” in the following). GO seq based Wallenius non-central hyper-geometric distribution, which could adjust for gene length bias, was implemented for GO enrichment analysis. KEGG (Kanehisa et al., 2008) is a database resource for understanding high-level functions and utilities of the biological system, such as the cell, organism, and ecosystem, from molecular-level information, especially large-scale molecular datasets generated by genome sequencing and other high-throughput experimental technologies (http://www.genome.jp/kegg/). We used KOBAS (Mao et al., 2005) software to test the statistical enrichment of the target gene candidates in KEGG pathways.

### Western Blot

The protein levels of *TMEFF1* and *GAPDH* were determined by western blotting. NAc tissues were lysed on ice using radioimmunoprecipitation assay lysis buffer (Solarbio, Beijing, China), and the total protein concentrations were established with a bicinchoninic acid protein assay kit (Solarbio, Beijing, China). The protein lysates were loaded and separated using sodium dodecyl–sulfate polyacrylamide gel electrophoresis before being moved to a polyvinylidene difluoride membrane. The blots were incubated with primary antibodies against TMEFF1 (1:1,000 dilution; sc-393 457; Santa Cruz Biotechnology, Dallas, TX, USA) and GAPDH (1:2,000 dilution; sc-47 724; Santa Cruz Biotechnology), then washed with Tris-buffered saline and incubated with IRDye 800CW goat anti-rabbit (LI-COR Biosciences, Lincoln, NE, USA) or IRDye 680RD goat anti-mouse (LI-COR Biosciences) secondary antibody for 1 hour at 37°C. The nitrocellulose membrane was then washed with Tris-buffered saline, and densitometric analysis was completed using appropriate channels (Odyssey; LI-COR Biosciences).

### Dual-Luciferase Reporter Assay

To verify if miR-592-3p could bind to the 3′-UTR of *TMEFF1* mRNA, HEK-293T cells were co-transfected with wild-type or mutant *TMEFF1* 3′-UTR plasmid in combination with an miR-592-3p mimic or miR-592-3p inhibitor; then, the wild-type or mutant *TMEFF1* 3′-UTR regions were inserted into the *Xho*I and *Not*I restriction sites of the psiCHECK-2 firefly luciferase vector (Geneseed Biotech, Guangzhou, China). Subsequently, the cells were gathered, and cell lysates were utilized to measure firefly and Renilla luciferase activities with a dual-luciferase reporter assay kit (Promega, Madison, WI, USA). In this context, the difference between the firefly and Renilla luciferase activities was calculated to determine the relative luciferase activity.

### Recombination Adeno-Associated Virus (rAAV) Constructs

The miR-592-3p/TMEFF1 downregulation or overexpression was achieved using a rAAV with a tetracycline-Off system (rAAV-CMV-tTA-WPRE pA) (Jiang et al., 2004). The rAAV-TRE-CMV-EGFP-miR-592-3p-WPRE-hGHpA (abbreviated as rAAV-miR-592-3p; titer exceeding 2.16 × 10^12^ vector genomes/mL) and rAAV-TRE-U6-Decoy RNA (miR-592-3p)-EGFP-SV40-pA (abbreviated as rAAV-Decoy-miR-592-3p; titer exceeding 4.13 × 10^12^ vector genomes/mL) were used to overexpress and knockdown miR-592-3p (BrainVTA, Wuhan, China), respectively. rAAV-TRE-CMV-TMEFF1-mCherry-WPRE pA (abbreviated as rAAV-TMEFF1; titer exceeding 5.20 × 10^12^ vector genomes/mL) and rAAV-TRE-U6-shRNA (TMEFF1)-CMV-mCherry pA (abbreviated as rAAV-shRNA-TMEFF1; titer exceeding 2.86 × 10^12^ vector genomes/mL) were used for the overexpression and knockdown of TMEFF1 (BrainVTA). The virus (0.2 μL) was injected using a calibrated glass microelectrode connected to a syringe pump (Hamilton) at a rate of 0.02 μL/min. Doxycycline (100 mg/L) was administered to mice via drinking water before rAAV injection and discontinued 24 hours before T2 test (WD13).

### Microinjection

Mice were anesthetized with isoflurane, then put in a stereotaxic apparatus. To conduct conditional gene interference disruption in the NAc, the packaged rAAV were microinjected into the bilateral NAc core (anteroposterior, +1.1 mm; mediolateral, ±1.0 mm; dorsoventral, −4.0 mm) or shell (anteroposterior, +1.1 mm; mediolateral, ± 0.8 mm; dorsoventral, −4.6 mm). After surgery, the mice were given 400 000 U/kg of penicillin per day for 3 days total to prevent infection and were provided free access to food and water. We verified the injection sites with fluorescent microscopy after completing behavioral tests; data were rejected when the injection site was off target.

### Statistical Analysis

Study data are expressed as mean ± SEM values. All statistical analyses were performed using a 1-way or 2-way ANOVA for each experiment, and Tukey’s test was applied to compare differences between 2 groups. All the statistical analyses were conducted using GraphPad Prism version 8 (GraphPad Software Inc., La Jolla, CA, USA). All *P* values <.05 were established as being statistically significant. The coding used to further break down *P* values was as follows: *P < .05, **P < .01, and ***P < .001.

## RESULTS

### Establishment of Cue-Induced Incubation Model of Morphine Craving

#### Between-Subjects Assessment

To establish the cue-induced CPP incubation model, 9 different groups were injected with saline or morphine (1 mg/kg or 5 mg/kg), and CPP test was conducted on withdrawal day 1 (WD 1/T1) and withdrawal day 14 (WD 14/T2) after the last training session (Fig. 1A). A 2-way ANOVA of between-subjects factors (i.e., morphine dose and test day) found a significant difference within morphine doses (*F*_(2,10)_ = 13.19, *P* = .003) and test days (*F*_(2,10)_ = 27.43; *P* < .001) as well as an interaction between morphine doses and test days (*F*_(4,17)_ = 8.24; *P* = .0017). Bonferroni’s multiple comparisons analysis showed that there was a significant difference between T2 and T1 in the 5-mg/kg morphine group (*P* < .01) but not in the 1-mg/kg morphine group (*P* > .05) (Fig. 1B).

#### Within-Subjects Assessment

Three different groups of mice were injected with either saline or morphine (1 mg/kg or 5 mg/kg) and continuously subjected to a CPP test on WD 1 and 14. A repeated-measures ANOVA confirmed a significant difference within morphine doses (*F*_(2,14)_ = 55.15; *P* < .001) and test days (*F*_(2,14)_ = 25.71; *P* < .001), and an interaction between morphine doses and test days (*F*_(4,14)_ = 14.59; *P* < .001). Bonferroni’s multiple comparisons analysis showed that there was a significant difference between T2 and T1 in the 5-mg/kg morphine group (*P* < .01) but not for the 1-mg/kg morphine group (*P* > .05) (Fig. 1C). The above results reveal that the morphine CPP score progressively increased following the start of withdrawal from the drug among mice exposed to 5 mg/kg of morphine but not in those exposed to 1 mg/kg, which successfully induced the incubation of morphine craving in the mice.

### Expression of miRNAs Involved in the Cue-Induced Incubation of Morphine Craving

#### miRNA Expression Profiling

To explore links between miRNA expression and the cue-induced incubation of morphine craving, the expression profile of miRNAs from the NAc tissue of CPP mice treated using 5 mg/kg of morphine at T1 and T2 was sequenced using RNA-seq. Compared with T1, 48 miRNAs were upregulated, and 52 miRNAs were downregulated at T2. All the differentially expressed miRNAs were showed in supplementary Table 2. The expression profiles of the changing miRNAs are presented in Figure 1D–E.

#### Candidate miRNA Selection

Of the 52 downregulated miRNAs, 8 miRNAs in the NAc (miR-126a-5p, miR-137-3p, miR-181c-5p, miR-186-5p, miR-592-3p, miR-29b-3p, miR-29c-3p, and miR-30e-5p) increased above 1.5-fold (*P* < .05) as verified by qRT-PCR. The miR-592-3p (*t* = 10.29, *P* < .05) had an approximately twofold change downregulated in the cue-induced incubation of morphine craving; therefore, we selected miR-592-3p as a candidate miRNA to further detect the molecular mechanisms involved in cue-induced incubation of morphine craving (Fig. 1F).

#### miR-592-3p Expression in the NAcC and NAcSh

Figure 1G shows the 2 subdivisions of the NAc: core (NAcC) (red arrows) and shell (NAcSh) (blue arrows). The expression of miR-592-3p was detected separately in NAcC and NAcSh with and without the CPP test. The results showed that the expression level of miR-592-3p at T2 was significantly decreased relative to T1 in NAcC (*t* = 25.28; *P* < .001) but not in NAcSh (*t* = .65, *P* > .05) (Fig. 1H); meanwhile, no significant difference was found without a CPP test (*P* > .05) in NAcC as well as NAcSh (Fig. 1I).

### miR-592-3p Played a Functional Role in Cue-Induced Incubation of Morphine Craving

**Figure 2.**
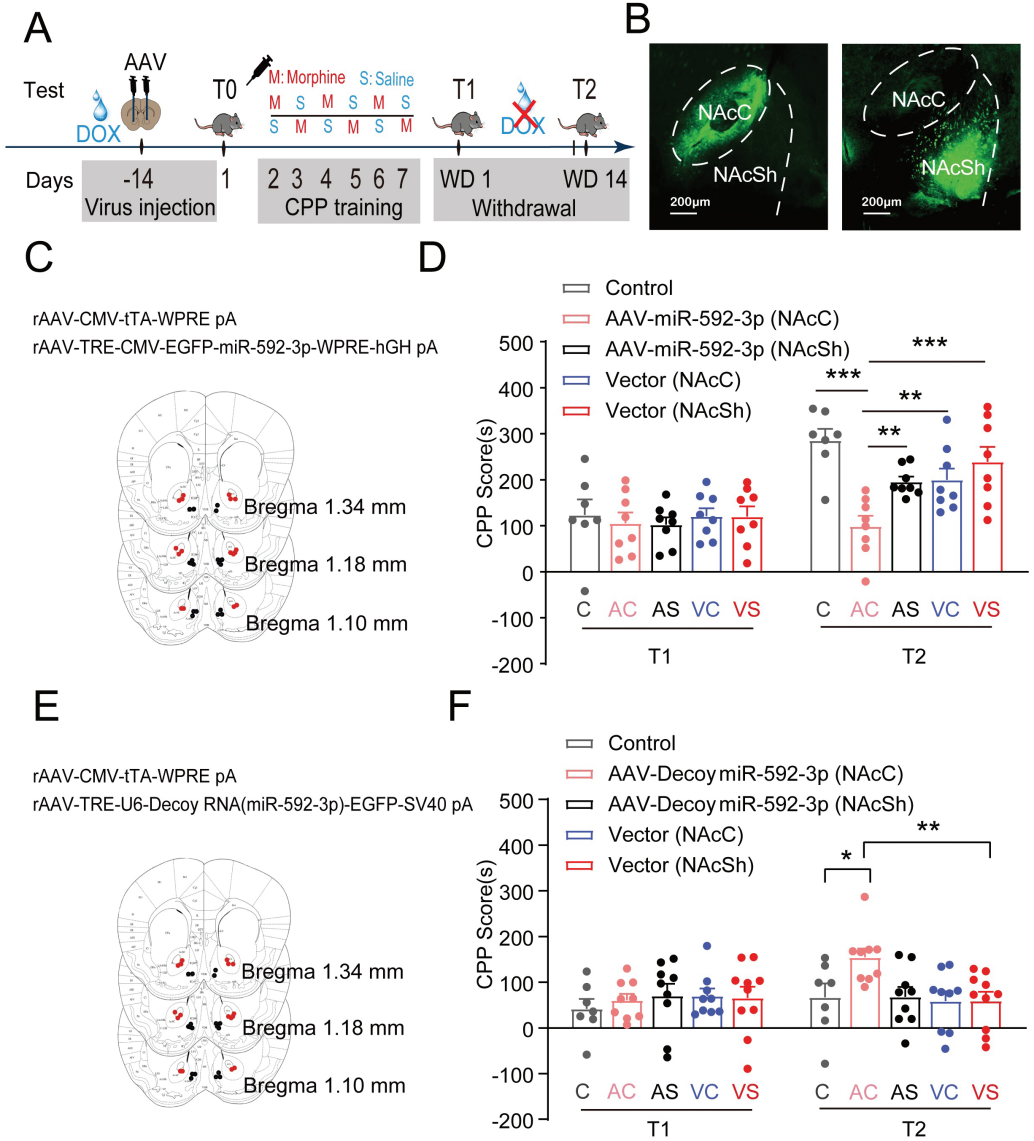
Expression of miR-592-3p in the cue-induced incubation of morphine craving. (A) Experimental timeline. (B) Schematics of the in vivo manipulation of the expression of viruses in the NAcC and NAcSh. (C) Schematics of the representative images of the overexpression viruses of miRNA-592-3p in the NAcC (red dots) and NAcSh (black dots). (D) The overexpression of miR-592-3p in the NAcC induced the CPP scores (5 mg/kg morphine conditioned) to decrease on T2 (C: control; AC: rAAV-Decoy miR-592-3p in NAcC; AS: rAAV-Decoy miR-592-3p in NAcSh; VC: vector in NAcC; VS: vector in NAcSh). ***P < .001 AC vs C, AC vs VS; **P < .01 AC vs AS, AC vs VC. (E) Schematics of the representative images of the knockdown viruses of miRNA-592-3p in the NAcC (red dots) and NAcSh (black dots). (F) Downregulation of miR-592-3p in the NAcC induced the CPP scores (1 mg/kg morphine conditioned) increased on T2. **P < .01 vs VS; *P < .05 vs C.

Next, gain- and loss-of-function of miR-592-3p strategies were applied to examine if miR-592-3p has a functional pivotal part in the process of cue-induced incubation of morphine craving. Figure 2A showed the timeline of rAAV (rAAV- miR-592-3p/rAAV-Decoy miR-592-3p) injection, gene expression manipulation, and behavioral tests. The rAAV injection site was verified by EGFP fluorescent protein (Fig. 2B,C,E), and miR-592-3p expression levels in the NAcC and NAcSh were verified by qRT-PCR. As shown in Figure 2D, CPP scores were analyzed using a repeated-measures ANOVA, including variables such as miR-592-3p expression and the withdrawal day. Post-hoc group differences suggested that overexpression of miR-592-3p by rAAV in the NAcC of 5 mg/kg morphine-conditioned mice led to a significant decrease in CPP scores in T2 (*P* < .001), whereas no difference in CPP scores was found in the NAcSh. Conversely, downregulation of miR-592-3p increased the CPP scores in T2 in the NAcC of 1 mg/kg morphine-conditioned mice (*P* < .05) but not in NAcSh (Fig. 2F). These results suggested that miR-592-3p has a functional part in the cue-induced incubation of morphine craving in the NAcC but not in the NAcSh.

#### Expression of TMEFF1 in Response to Cue-Induced Incubation of Morphine Craving

**Figure 3.**
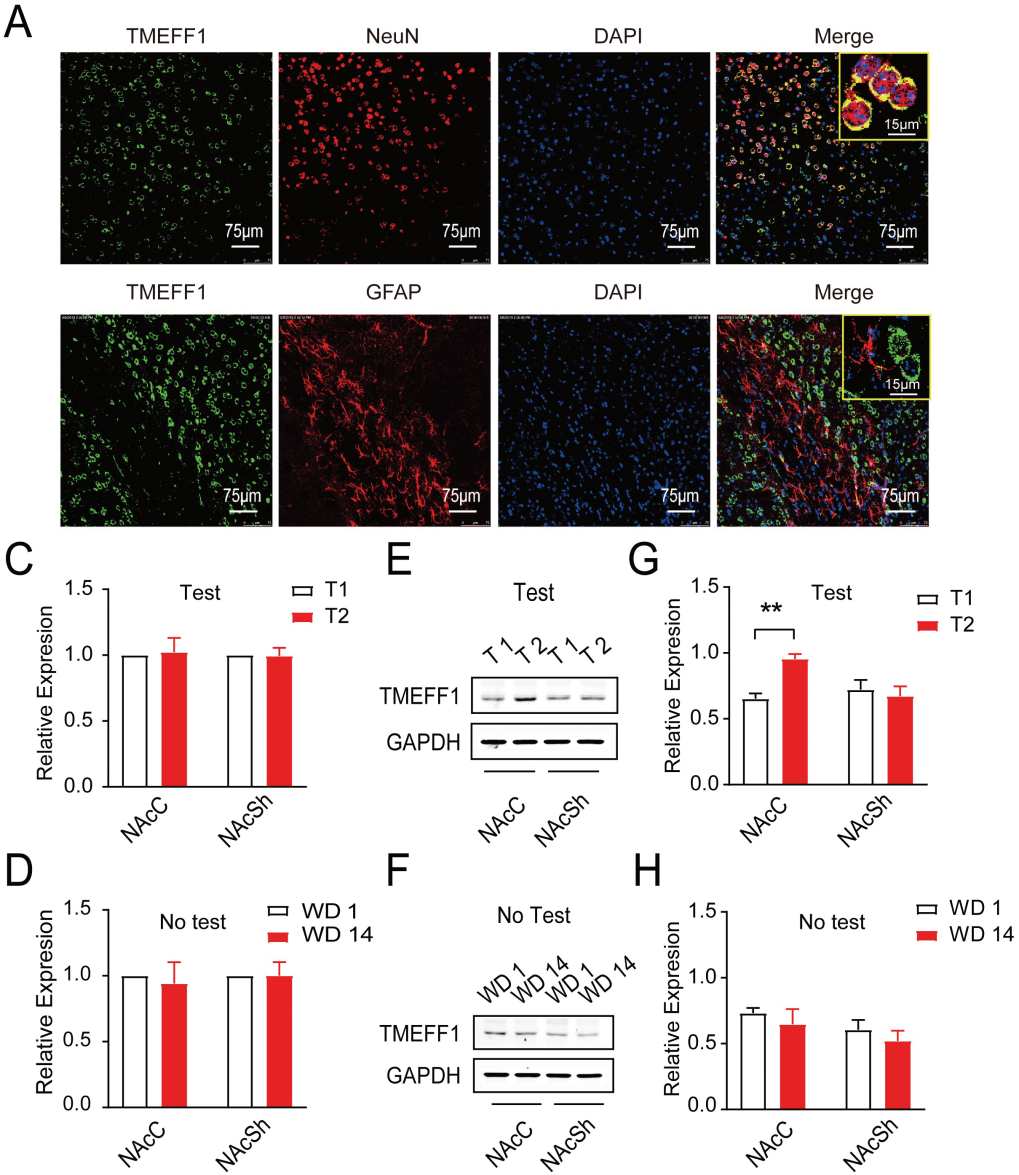
Expression of TMEFF1 in response to incubation of morphine craving. (A, B) The subcellular localization of TMEFF1 by using the laser scanning microscope. The TMEFF1 protein labeled with green fluorescence, A: neuron nuclei (NeuN), and B: astrocytes (GFAP) labeled with red fluorescence (Scale bar = 75 μm). (C) The expression of *TMEFF1* mRNA between T1 and T2 in NAcC or NAcSh. (D, E) Western-blot analysis of TMEFF1 and GAPDH expression between T1 and T2 in NAcC or NAcSh. **P < .01, vs T1 in NAcC. (F) The expression of *TMEFF1* mRNA between WD1 and WD2 in NAcC or NAcSh without test. (G, H) Western-blot analysis of *TMEFF1* and GAPDH expression between WD1 and WD2 in NAcC or NAcSh without the test.

MiRNAs are critical for attenuating the stability and translation of mRNAs via base pairing to partially complementary sites in the 3’-UTR of their target genes. To further analyze the biological functions of miR-592-3p in the cue-induced incubation of morphine craving, TargetScan8.0 and miRanda were used to predict the target genes of miR-592-3p. Among the computationally predicted targets, *TMEFF1* was indicated for further validation owing to its higher predictive scores and effects on regulatory factors of addiction-related proteins (supplementary Table 3), and a complementary sequence of miR-592-3p was identified in the 3’-UTR of *TMEFF1*. The subcellular localization of *TMEFF1* was observed using a laser scanning microscope, and *TMEFF1* was mainly found to be localized to the neuron membrane (Fig. 3A–B). As shown in Figure 3C, there was no significant difference in *TMEFF1* mRNA level between T1 and T2 both in NAcC (*t* = .193, *P* > .05) and NAcSh (*t* = .090, *P* > .05). However, we discovered that the expression level of TMEFF1 protein in T2 was significantly higher than that in T1 in the NAcC (*t* = 7.291; *P* < .01), while no significant difference was determined in the NAcSh (*t* = 1.837; *P* > .05) (Fig. 3D–E). Conversely, no differential expression of *TMEFF1* mRNA (Fig. 3F) or protein (Fig. 3G–H) in either the NAcC or NAcSh in any test was noted (*P* > .05). These results suggested that TMEFF1 protein was highly expressed in the cue-induced incubation of morphine craving in the NAcC.

### Function of TMEFF1 in Response to Cue-Induced Incubation of Morphine Craving

**Figure 4.**
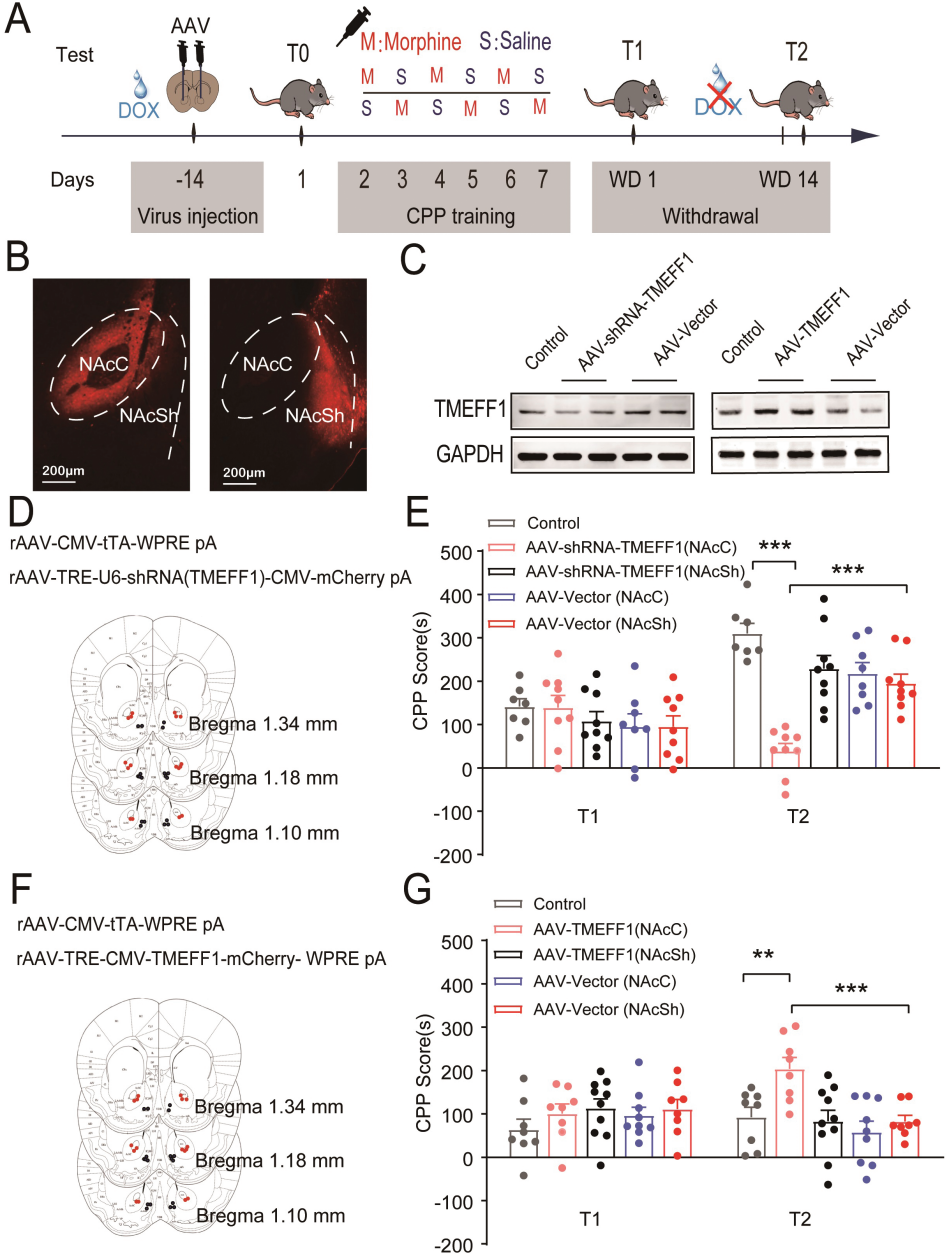
Function of *TMEFF1* in response to cue-induced incubation of morphine craving. (A) Experimental timeline. (B) Schematics of the in vivo manipulation and a representative image of the expression of viruses in the NAcC and NAcSh. (C) The expression of TMEFF1 was decreased or increase after injection of rAAV-shRNA-TMEFF1 or rAAV-TMEFF1 by western-blot analysis. (D) Schematics of the representative images of the injection of the knockdown viruses of TMEFF1 (rAAV-TRE-U6-shTMEFF1-CMV-mCherry pA) in the NAcC (red dots) and NAcSh (black dots). (E) Downregulation of TMEFF1 in the NAcC induced the CPP scores decreased on T2 in 5 mg/kg morphine conditioned mice. ***P < .001 vs control and rAAV-vector. (F) Schematics of the representative images of the injection of overexpression viruses of TMEFF1 (rAAV- TRE-CMV-TMEFF1-mCherry-WPRE pA) in the NAcC (red dots) and NAcSh (black dots). (G) The overexpression of TMEFF1 in the NAcC induced the CPP scores increased on T2 in 1 mg/kg morphine-conditioned mice. ***P < .001 vs control, ***P < .001 vs rAAV-vector.

At this point, to examine whether *TMEFF1* has a functional part in the process of cue-induced incubation of morphine craving, gain- and loss-of-function analyses of *TMEFF1* strategies were performed. Two weeks before the T0 test, rAAV for downregulating (rAAV-shRNA-TMEFF1) or overregulating (rAAV-TMEFF1) the expression TMEFF1 were microinjected into the bilateral NAcC or NAcSh separately. Prior to the injection of rAAV virus, 100 mg/L doxycycline was administered to mice via drinking water and discontinued 24 hours before T2 (Fig. 4A). The rAAV injection site was verified by the mCherry fluorescent protein (Fig. 4B,D,F). Thereafter, western-blot results showed that rAAV-shRNA-TMEFF1 or rAAV-TMEFF1 led to a decrease or increase in TMEFF1 levels compared with the control and rAAV-vector (Fig. 4C).

As shown in Figure 4E, the downregulation of TMEFF1 in the NAcC may significantly reduce the morphine CPP scores at T2 in contrast to the control (*P* < .001) and rAAV-Vector (*P* < .001) groups (5 mg/kg morphine conditioned), while the overexpression of TMEFF1 can lead to significantly increased morphine CPP scores at T2 (1 mg/kg morphine conditioned) (*P* < .01) (Fig. 4G). The regulation of TMEFF1 expression by rAAV did not affect morphine CPP scores on T1, whether in NAcC (*P* > .05) or NAcSh (*P* > .05). These results indicate that TMEFF1 positively regulates the cue-induced incubation of morphine craving in the NAcC, indicating that TMEFF1 has a functional part in the cue-induced incubation of morphine craving.

### miR-592-3p Regulated Expression of TMEFF1 in NAcC During Cue-Induced Incubation of Morphine Craving

**Figure 5.**
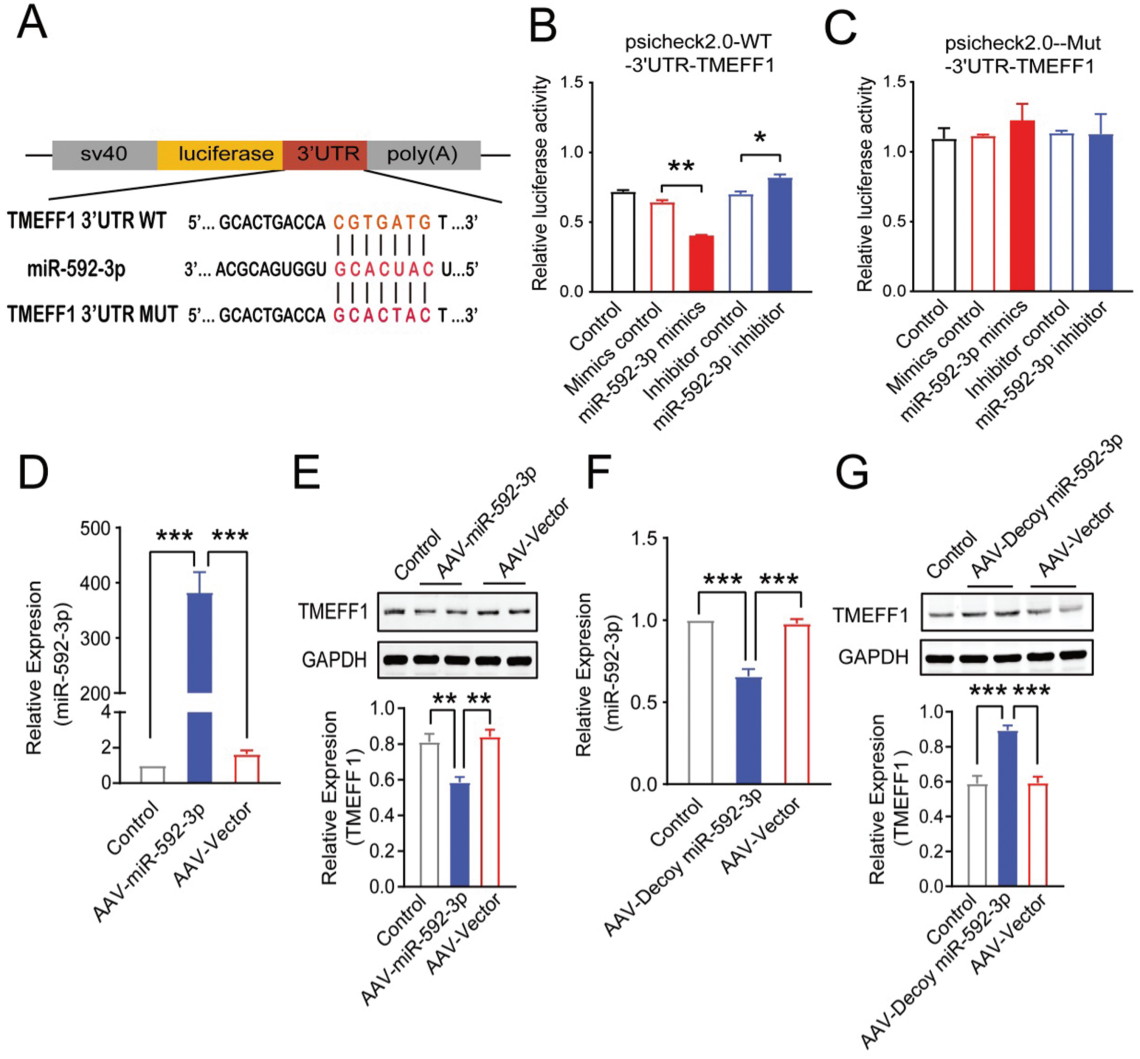
The expression of TMEFF1 was regulated by miR-592-3p in the NAcC. (A) The predicted miR-592-3p binding site in the 3′-UTR region of TMEFF1 and the designed mutant sequences of TMEFF1 were indicated. (B, C) Relative luciferase activities of miR-592-3p were measured in the 293T cells that co-transfected TMEFF1 with wild-type luciferase vector or mutant luciferase vector, ** *P* < .01 vs mimics control, * *P* < .05 vs inhibitor control. (D) The expression of miR-592-3p was significantly increased after injection of rAAV-miR-592-3p (rAAV-TRE-CMV-EGFP-miR-592-3p-WPRE-hGHpA), ***P < .001 vs control or rAAV-vector (n = 4). (E) The expression of TMEFF1 was significantly decreased after injection of the upregulated rAAV-miR-592-3p during the incubation of morphine craving. **P < .01 vs control or rAAV-vector (n = 6). (F) The expression of miR-592-3p was significantly decreased after injection of rAAV-Decoy-miR-592-3p (rAAV-TRE-U6-Decoy RNA (miR-592-3p)-EGFP-SV40-pA), ***P < .001 vs control or rAAV-vector (n = 4). (G) The expression of TMEFF1 was significantly increased after injection of down-regulation rAAV-Decoy-miR-592-3p during the incubation of morphine craving. ***P < .001 vs control or rAAV-vector (n = 6).

Bioinformatic analysis results support the idea that the 3′-UTR of *TMEFF1* mRNA has a single highly conserved potential binding site of miR-592-3p (Fig. 5A). The direct binding of miR-592-3p to the 3′-UTR of the *TMEFF1* was examined by generating luciferase reporter plasmids that contained the miR-592-3p–binding site or mutant sequence. As anticipated, the miR-592-3p mimic led to significant reductions in luciferase activity relative to the negative mimic control (*P* < .01), while the inhibition of miR-592-3p elevated the luciferase activity (*P* < .05) (Fig. 5B); nevertheless, the mutated sequence of the *TMEFF1* 3′-UTR abrogated the repressive effects of miR-592-3p on the luciferase activity of its target 3′-UTR (Fig. 5C). These results indicate that miR-592-3p directly binds to the 3′-UTR of the *TMEFF1* mRNAs.

To determine if miR-592-3p regulates the translation of TMEFF1 protein, miR-592-3p was overexpressed by injecting rAAV into NAcC, and the results of the real-time PCR revealed that rAAV-miR-592-3p leads to a significant elevation in miR-592-3p levels in contrast to the control (*P* < .001) and rAAV-vector (*P* < .001) groups (Fig. 5D). Meanwhile, the western-blot results revealed that rAAV-miR-592-3p overexpression could induce a decrease in TMEFF1 protein levels compared with the control (*P* < .01) and rAAV-vector (*P* < .01) (Fig. 5E) in the incubation of morphine craving. In addition, the rAAV-Decoy-miR-592-3p was injected to test the effects of the downregulated expression of miR-592-3p in NAcC. Here, the results of real-time PCR revealed that rAAV-Decoy-miR-592-3p application can trigger a significant decrease in miR-592-3p levels in contrast to the control (*P* < .001) and rAAV-Vector (*P* < .001) groups (Fig. 5F). Western-blot results revealed that downregulation of rAAV-Decoy-miR-592-3p could induce the increase in the TMEFF1 protein levels in contrast to the control (*P* < .001) and rAAV-vector (*P* < .001) (Fig. 5G). These results suggested that miR-592-3p regulated the expression of TMEFF1 in the NAcC during the cue-induced incubation of morphine craving.

## DISCUSSION

Following prolonged withdrawal from drug self-administration in laboratory animals, cue-induced drug-craving progressively increases, a behavioral phenomenon known as “incubation of drug craving” (Li et al., 2015). This “incubation” of the drug craving phenomenon may be a key factor in the persistence of psychological addiction. The epigenetic factor is a novel approach to examine the underlying mechanisms of the pathogenesis of the incubation of drug craving (Werner et al., 2021). MiRNAs, as epigenetic modulators, might have a part in this drug-craving process. The overall goal of the current work was to determine if miRNAs mediate neurobiological mechanisms that are responsible for the cue-induced incubation of morphine craving. In this work, small RNA-seq was performed to analyze the miRNA expression profile from NAc tissues in the incubation of morphine craving mice model, and we identified that miR-592-3p is abnormally downregulated. Loss of miR-592-3p expression resulted in an increase in TMEFF1 levels in NAcC, which in turn led to cue-induced incubation of morphine craving, whereas overexpression of miR-592-3p resulted in a decrease in TMEFF1 levels in NAcC and reduced the cue-induced incubation of morphine craving.

The incubation of cravings has been broadly examined in the drug self-administration paradigm, but not in the CPP paradigm (Nicolas et al., 2019), yet the utilization of the CPP paradigm to reflect the incubation of cravings has already been proposed by Li et al. (Li et al., 2008), who revealed that the CPP scores affiliated with low doses of morphine (1 mg/kg and 3 mg/kg) but not high doses (10 mg/kg) increased progressively throughout the first 14 days of abstinence in rats. The increased CPP scores concurrent with the low doses of morphine were utilized to reflect incubated craving. According to our previous study (Yu et al., 2021), morphine doses of 1, 5, and 10 mg/kg were used to establish the cue-induced CPP incubation of the morphine craving model in mice. Identically, our results revealed that the CPP score of the dose of morphine at 5 mg/kg increased progressively during WD 14, which successfully induced the incubation of morphine craving in mice. Different animal models of morphine incubation might result in a different dose of morphine. In our study, the dose of 5 mg/kg morphine incubation model was observed in mice and the dose of 1 mg/kg and 3 mg/kg morphine was used in rats in the incubation model in Li’s research. CPP scores in concert with low doses of morphine (5 mg/kg) increased progressively during the abstinence period, like the increase in active responses in the self-administration paradigm. These results suggest that the incubation of the craving phenomenon could be modeled by the CPP paradigm.

The role of miRNAs in response to the incubation of drug craving remains largely unknown. Here, we first screened alterations in miRNAs associated with the incubation of morphine craving, and, among such miRNAs, miR-592-3p was of particular interest because it demonstrated the most dramatic change in expression in the NAc during the incubation of morphine craving in model mice. This catalog of morphine-regulated miRNAs can offer new insight into the molecular mechanisms of the incubation of morphine craving and serve as a resource for future studies. miRNAs, which influence the interactions among these molecules and their targets in different diseases, such as addiction, may serve as valuable targets for more efficient therapies (Dreyer, 2010; Pietrzykowski, 2010; Bali and Kenny, 2013; Bhaskaran et al., 2019). Chandresekar and Dreyer have shown that the miRNAs miRNA-124a and let-7d were downregulated in the striatum in response to cocaine treatment (Chandrasekar and Dreyer, 2011). Later, Rodriguez found that the miR-133b target PITX3 may be a mechanism for the development of an addiction to morphine or other drugs able to increase dopaminergic levels in the extracellular space that are abused (Rodriguez, 2012). Other studies have also suggested the involvement of several miRNAs (miR-212, miR-132, miR-181a, miR-140, miR-190, and so on) in the dendritic spine morphogenesis and the development of addiction (Chandrasekar and Dreyer, 2011; Bali and Kenny, 2013; Smith and Kenny, 2018). Specific miRNAs have emerged in the literature as key regulators leading to addiction and could serve as valuable targets for more effective therapies. In the present work, we confirmed that miR-592-3p was decreased in the incubation of morphine craving, and gain- and loss-of-function results pertaining to miR-592-3p revealed that miR-592-3p has a functional part in cue-induced incubation of morphine craving in the NAc. These results support the idea that a set of miRNAs have an important part in the morphine-addiction process.

To understand the mechanism of miR-592-3p, one must first determine its target genes and binding sites. TargetScan and miRanda analyses indicated that TMEFF1 may be a potential target of miR-592-3p. TMEFF1, a transmembrane protein, appeared to be highly expressed in the human embryo and nerve tissues (Kanemoto et al., 2001). Prior studies have primarily focused on its role in the middle/late developmental stage of the embryo and the effect of its regulation on the central nervous system (Kanemoto et al., 2001; Nie et al., 2019; Wang et al., 2020). Listos et al. revealed that TMEFF1 is a carcinogenic gene in ovarian cancer that can be regulated via the MAPK and PI3K/AKT signaling pathways, which are involved in morphine addiction (Listos et al., 2019). TMEFF1 was originally discovered to be differentially expressed in brain tissues. Our study found that TMEFF1 was mainly expressed in the neuron membrane. As the target gene of miR-592-3p, we verified the content and function of TMEFF1 in the cue-induced incubation of the morphine craving model. Our results revealed that the expression of TMEFF1 at protein levels was significantly upregulated in the cue-induced incubation of morphine craving in the NAcC. Furthermore, our results showed that downregulation of TMEFF1 could significantly decrease the morphine incubation, and its overexpression significantly increased morphine incubation, suggesting a functional role of TMEFF1 in cue-induced incubation of morphine craving. Our results revealed that overexpression of miR-592-3p decreased TMEFF1 protein levels in the incubation of morphine craving and its downregulation led to a significant increase in TMEFF1 protein levels, which indicated that miR-592-3p has a functional part in the regulation of TMEFF1 expression during the incubation of morphine craving. Considering the ceiling effect on CPP scores of 5-mg/kg morphine conditioned mice in T2, 1 mg/kg morphine was used in the experiments, and we found that the downregulation of miR-592-3p and overexpression of TMEFF1 increased the CPP scores of T2 and facilitated the cue-induced incubation of morphine craving. Therefore, our results provide new insights into functions of TMEFF1 in the brain.

The incubation of drug craving involves neuroadaptations in the circuitry underlying reward and motivation (Wolf, 2016). NAc has a crucial part in the brain’s system of reward, motivation, aversion, and reinforcement learning; hence, it is associated with addiction (Klawonn and Malenka, 2018). NAc is considered a part of the basal ganglia and is divided into 2 anatomical components, the shell (NAcSh) and core (NAcC), each having its functions. The NAc core and shell are heterogeneous structures that may be distinguished according to morphology and projections. This neuroanatomical evidence has led to the conduct of many studies on the role of NAc core and shell in motivated behaviors and how drugs affect the reward system in conditioned and unconditioned circumstances (Glass et al., 2008; Ito and Hayen, 2011; West and Carelli, 2016). In our study, the expression of miR-592-3p was significantly decreased and had a functional role in cue-induced incubation of morphine craving in the NAcC, but not in NAcSh. Additionally, gain- and loss-function analyses showed that miR-592-3p has a functional part in the regulation of TMEFF1 expression in the NAcC. Wang et al. suggested that the NAc core and shell lesions elicited dissociable effects on the reactivation of extinguished CPP, postulating that the NAc core might play a more important role in resisting the reactivation of extinguished CPP in morphine-addicted rats (Wang et al., 2008). Overall, these results support the existence of an important role for the NAc core in the incubation of morphine craving.

The main strength of this study can be summed up as follows: a gain- and loss-of-function strategy was applied to understand the mechanism of miR-592-3p and its target gene, *TMEFF1*, in morphine craving incubation, which enhances the regulation result of miR-592-3p and *TMEFF1*. However, some limitations should also be noted. First, although some reports and our data showed that the CPP paradigm could be a well-defined model for the phenomenon of craving incubation, the paradigm of self-administration has been more widely studied to date in the incubation of drug craving. Future studies with a self-administration paradigm should be done to verify the role of miR-592-3p and its target gene, *TMEFF1*, during incubation of morphine craving. Secondly, the function of miR-592-3p and TMEFF1 was studied respectively, co-regulation of miR-592-3p and *TMEFF1* by rescue assay warrants further studies. Last, we only highlighted the role of miR-592-3p and its target gene, *TMEFF1*. However, other target genes require further study. Thorough analysis of these target genes could further our knowledge of the molecular mechanisms underlying the incubation of morphine craving.

## Conclusions

In conclusion, the expression of miR-592-3p was downregulated in the process of cue-induced incubation of morphine craving. MiR-592-3p has a crucial part in the incubation of morphine craving by post-transcriptional regulation of TMEFF1. Furthermore, upregulation of miR-592-3p improves morphine craving. The results of this work offer evidence to help determine the underlying mechanism of the incubation of morphine craving and may pave the way for important new avenues for preventing drug relapse.

## Supplementary Material

pyac004_suppl_Supplementary_Table_S1

pyac004_suppl_Supplementary_Table_S2

pyac004_suppl_Supplementary_Table_S3
